# A Multi-Omics Protocol for Swine Feces to Elucidate Longitudinal Dynamics in Microbiome Structure and Function

**DOI:** 10.3390/microorganisms8121887

**Published:** 2020-11-28

**Authors:** Laurin Christopher Gierse, Alexander Meene, Daniel Schultz, Theresa Schwaiger, Claudia Karte, Charlotte Schröder, Haitao Wang, Christine Wünsche, Karen Methling, Bernd Kreikemeyer, Stephan Fuchs, Jörg Bernhardt, Dörte Becher, Michael Lalk, KoInfekt Study Group, Tim Urich, Katharina Riedel

**Affiliations:** 1 Institute of Microbiology, University of Greifswald, Felix-Hausdorff-Str. 8, 17489 Greifswald, Germany; laurin.gierse@uni-greifswald.de (L.C.G.); alexander.meene@uni-greifswald.de (A.M.); haitao.wang@uni-greifswald.de (H.W.); christine.wuensche@uni-greifswald.de (C.W.); joerg.bernhardt@uni-greifswald.de (J.B.); doerte.becher@uni-greifswald.de (D.B.); 2 Institute of Biochemistry, University of Greifswald, Felix-Hausdorff-Str. 4, 17489 Greifswald, Germany; daniel.schultz@uni-greifswald.de (D.S.); methling@uni-greifswald.de (K.M.); lalk@uni-greifswald.de (M.L.); 3 Friedrich-Loeffler-Institut, Greifswald-Insel Riems, Südufer 10, 17493 Greifswald, Germany; theresa.schwaiger@boehringer-ingelheim.com (T.S.); claudia.karte@fli.de (C.K.); charlotte.schroeder@fli.de (C.S.); 4 Institute for Medical Microbiology, Virology and Hygiene, Rostock University Medical Centre, Schillingallee 70, 18055 Rostock, Germany; bernd.kreikemeyer@med.uni-rostock.de; 5 Division of Nosocomial Pathogens and Antibiotic Resistance, Robert Koch Institute Wernigerode, Burgstraße 37, 38855 Wernigerode, Germany; fuchss@rki.de

**Keywords:** biomedical model swine, gastrointestinal microbiome, integrated multi-omics, 16S rRNA gene-sequencing, metaproteomics, metabolomics

## Abstract

Swine are regarded as promising biomedical models, but the dynamics of their gastrointestinal microbiome have been much less investigated than that of humans or mice. The aim of this study was to establish an integrated multi-omics protocol to investigate the fecal microbiome of healthy swine. To this end, a preparation and analysis protocol including integrated sample preparation for meta-omics analyses of deep-frozen feces was developed. Subsequent data integration linked microbiome composition with function, and metabolic activity with protein inventories, i.e., 16S rRNA data and expressed proteins, and identified proteins with corresponding metabolites. 16S rRNA gene amplicon and metaproteomics analyses revealed a fecal microbiome dominated by *Prevotellaceae*, *Lactobacillaceae*, *Lachnospiraceae*, *Ruminococcaceae* and *Clostridiaceae.* Similar microbiome compositions in feces and colon, but not ileum samples, were observed, showing that feces can serve as minimal-invasive proxy for porcine colon microbiomes. Longitudinal dynamics in composition, e.g., temporal decreased abundance of *Lactobacillaceae* and *Streptococcaceae* during the experiment, were not reflected in microbiome function. Instead, metaproteomics and metabolomics showed a rather stable functional state, as evident from short-chain fatty acids (SCFA) profiles and associated metaproteome functions, pointing towards functional redundancy among microbiome constituents. In conclusion, our pipeline generates congruent data from different omics approaches on the taxonomy and functionality of the intestinal microbiome of swine.

## 1. Introduction

The microbiome of the human intestinal tract is considered one of the main drivers for host fitness. It is involved in processes such as immune system regulation [1], nutrient utilization [2], and maintenance of intestine function [3]. Changes in the human microbiome were linked to diseases such as obesity [4], type-2 diabetes [5], colorectal cancer [6], Crohn’s disease or inflammatory bowel syndrome [7,8]. Furthermore, an extrinsic short-term disturbance in the intestinal homeostasis, e.g., by antibiotic treatment, can cause long term disturbance within the commensal intestinal microbiome in a porcine model [9]. Dietary changes also shape the composition of the microbiome [10,11]. Comparison between high-fat/low-fiber (HF) and low-fat/high-fiber (LF) diets revealed a stimulating effect by LF diet on beneficial bacteria (such as *Bifidobacterium* spp. and *Lactobacillus* spp.) and the production of short-chain fatty acids (SCFAs). HF content was associated with bacterial groups (e.g., *Enterobacteriaceae*) with negative impact on human health status, e.g., *Escherichia coli* and *Salmonella enterica*.

Addressing the adaptation processes of the human microbiome within the intestinal environment during an infection paves the way to understanding the metabolic impact caused by bacterial and/or viral pathogens. To analyze these changes, there is urgent need for an appropriate in vivo model. Established animal models mainly focus on mice or rats. In particular, mice are widely used as the infection model for the human situation, but the use of this model is hampered by differences between humans and mice in physiology as well as in innate and adaptive immune systems. By comparison of murine and human gastrointestinal anatomy, differences become apparent, for instance the murine cecum is relatively large compared to that of humans. The colon of mice is rather smooth, not divided into different sections (ascending, transverse and descending colon) with a thin muscularis mucosae and without haustrum and taenia coli, in contrast to the human colon [12,13]. It is not only the differing body sizes and lifespans that trigger these differences, but also the occupancy of different ecological niches, resulting in evolution in different environments. Thus, interpretation of preclinical data obtained in mice is difficult [14]. Due to these limitations, alternative model systems to analyze the importance of the intestinal microbiome are required.

Pigs are becoming a popular alternative model organism for research on human health and disease [15]. This is explained by the large similarity between swine and humans in organ size, physiology, genetics, and immune cell populations [15,16]. Recently, a swine model with human microbiota was developed to better reflect the human gut [17]. Furthermore, pigs have a typical mammalian composition of the intestinal microbiome dominated by Firmicutes and Bacteroidetes, resembling that of humans [18,19]. By taking a deeper look on genus level, the intestinal tract of swine and human share several bacterial groups, such as *Prevotella, Faecalibacterium, Coprococcus, Streptococcus, Megasphaera, Dialister* and *Subdoligranulum*, which were absent or very low abundant in the murine intestinal tract [19,20,21]. In contrast to that, the study of Ley and colleagues of the murine intestinal tract demonstrated that 85% of the observed bacterial genera were not detected in humans [22]. Dominant and highly prevalent genera of the murine intestinal tract were, for example, *Anaerostipes*, *Parabacteroides* and *Eggerthella* [23]. Higher abundant in mice, but nevertheless prevalent in humans were, for example, *Lactobacillus*, *Turicibacter* and *Alistipes* [13].

Within recent years multi-omics approaches have been applied to answer various scientific questions [24,25,26]. However, to the best of our knowledge, such an integrated approach has not been used to analyze the gastrointestinal microbiome of swine. Recently, different studies focusing on single omics-approaches to elucidate the intestinal microbiota of swine, e.g., by 16S rRNA gene sequencing [10,18], have been published. Tröscher-Mußotter and colleagues published the first metaproteome analysis highlighting the differences between sections from the small and large intestine of swine [27]. Furthermore, they showed that the relative abundance of proteins involved in energy production and conversion was higher in mucosa than digesta samples. In contrast, proteins involved in lipid transport and metabolism, and short-chain fatty acid production were more abundant in the digesta sample. In spite of that, Tröscher-Mußotter et al. suggested that an integrative approach combining other omics methods would facilitate the understanding of the intestinal microbiome [27]. Combination of metaproteomic and metabolomic data allows the prediction of active metabolic pathways [28]. Moreover, metaproteomics have been employed to elucidate the influence of biological factors including disease on taxonomy and function of the microbiome [29,30,31]. Integrating all the available comprehensive multi-omics technologies constitutes a powerful and promising approach to investigate structure and functionality of gut microbiomes, and provides new insights into host–microbiome relationships within this complex environment that remain hidden when targeting only one single type of molecule.

In the presented study three healthy swine, which served as control group for a 30 day influenza A virus H1N1 infection, were evaluated as baseline for its potential as a biomedical model. Inter-individual variance was of special interest as it could hide infection caused shifts in the respiratory as well as intestinal microbiomes. Our study aimed to (1) establish a standardized, reproducible multi-omics pipeline, including 16S rRNA gene sequencing, metaproteomics, and metabolomics analysis, (2) to characterize and monitor the temporal structure and function of the intestinal microbiome of the uninfected animals. Subsequently, this pipeline was used to elucidate the interindividual variability of healthy animals together with the time-dependent progression of the microbiome. Established methodologies and knowledge on the natural development of the swine intestinal microbiome during the experimental time-frame are crucial for further studies elucidating the impact of infections, as well as other interventions or events in the pig’s life, on the swine gastrointestinal microbiome.

## 2. Materials and Methods

### 2.1. Animal Study Design

**Figure 1:**
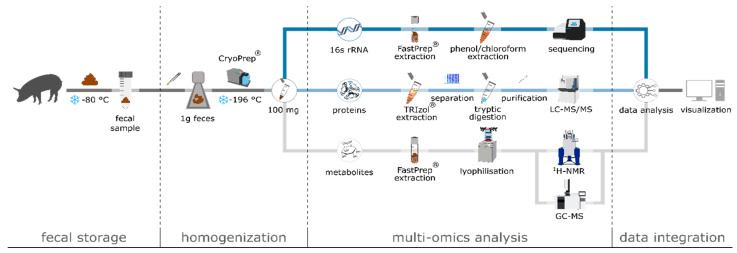
Integrated multi-omics workflow for 16S rRNA gene sequencing (blue), metaproteomics (light blue) and metabolomics (grey) out of the same fecal sample. After sampling fecal material was immediately placed on dry ice and stored at −80 °C, followed by CryoPrep-based homogenization, optimized target-molecule extraction, and analysis, data analysis, integration and visualization.

All samples for this study were provided by the Department of Experimental Animal Facilities and Biorisk Management of the Friedrich-Loeffler-Institut on the Isle of Riems within the H1N1pdm09 animal experiment with the reference number 7221.3-1-035/17 [32]. For this study, three mock-infected German landrace pigs, which were eight weeks of age at the beginning of the study, were analyzed. From the third day of their lifes all animals were fed the prestarter diet OlymPig (Agravis, Münster, Germany), for four weeks, additionally to the mother’s milk. At the age of four weeks, they were weaned and received a mixture of the OlymPig prestarter and PANTO start (Hamburger Leistungsfutter GmbH, Hamburg, Germany) diet. Afterwards, the animals were fed the PANTO start diet only (24 days before the first sampling day). Both diets were wheat, barley and soy based. Feces were collected from three healthy swine. The sampling scheme is shown in Table 1. Individual fecal samples were collected during or within less than 30 seconds (s) after defecation over a time period of 30 days to cover longitudinal shifts (see feces homogenization and splitting procedure in Figure 1). Furthermore, digesta from intestinal sections (IS) (ileum, proximal and distal colon from three animals) were sampled during necropsy on day 30. All samples were immediately frozen on dry ice and subsequently stored at −80 °C.

### 2.2. Sample Processing

The deep-frozen samples were cut with a sterile scalpel to approximately 1 g pieces and placed into the middle of a Covaris^®^ Tissue Tube™ TT1. To keep the fecal samples deep-frozen the tissue tube was then placed into liquid nitrogen for 60 s. The Covaris^®^ CP02 CryoPrep™ instrument (Covaris Ltd., Brighton, UK) was set to impact level 5 and the loaded tissue tube was treated two times with liquid nitrogen for 30 s between the homogenization steps. The resulting fecal powder was then used for the three different omics-analyses described below.

### 2.3. DNA Extraction, 16S rRNA Gene Amplicon Library Preparation, Sequencing, and Bioinformatic Processing

Nucleic-acids were extracted from the fecal powder by a bead beating phenol-chloroform extraction protocol [33], followed by nucleic acid precipitation with 3 M Na-acetate and isopropanol. After washing with 70% *v*/*v* ethanol the resulting DNA pellets were resuspended in DEPC-treated MilliQ water for downstream applications. DNA content was quantified via Qubit^TM^ dsDNA Broad Range Assay Kit (Invitrogen^TM^). DNA was diluted to 5 ng/µL for PCR. Amplicon and Index-PCR were performed using the V4 primer pair 515F (5′-GTG-YCA-GCM-GCC-GCG-GTA-A-3′)/806R (5′-GGA-CTA-CNV-GGG-TWT-CTA-AT-3′) [34,35] followed by a PCR clean up between and after both amplifications with AMPure XP beads. Libraries were quantified via Invitrogen^TM^ Qubit^TM^ dsDNA broad-range assay kit, normalized to a final concentration of 5 pM, denatured with NaOH and sequenced via Illumina MiSeq with an approximate output from 75,000 to 132,600 reads per sample. The sequences were submitted to European Nucleotide Archive (ENA), with the project number PRJEB39963, accession number ERP123542 and the project name “KoInfekt-multi-omics-pipeline-swine”. Resulting 16S rRNA gene sequences were processed in R [36] using the dada2 pipeline-package version 1.11.1 (R-version 3.6.1). Paired-end reads were truncated to a final length of 240 bp each with a minimal overlap of 50 bp. Quality-filtered 16S rRNA gene sequences (maxN = 0, maxEE = 2, and truncQ = 2) were clustered to amplicon sequence variants (ASVs), a higher resolution analog of operational taxonomic units (OTUs) [37]. Chimeras were removed and ASVs were assigned via SILVA 132 database [38]. ASVs classified as chloroplasts or mitochondria were removed afterward. Advanced bioinformatic processing (e.g., alpha- and beta diversity, Bray-Curtis dissimilarities and non-metric multidimensional scaling) was performed in R using “vegan”, “ggplot”, “phyloseq”, ”plyr”, “reshape2” packages. For statistical analysis, PERMANOVA test was performed (*p* = 0.05), using the package “vegan”.

### 2.4. Protein Extraction and Preparation for Mass Spectrometry

1 mL TRIzol Reagent (Invitrogen, Carlsbad, CA, USA) was added to 100 mg of the homogenized fecal sample and mixed 3 × 30 s on a vortex for resuspension. Afterwards, the manufacturer’s protocol based on Chomczynski, P. [39] was performed, which comprises the following steps: incubation for 5 minutes (min), addition of 0.2 mL chloroform, incubation for 3 min followed by centrifugation for 15 min at 12,000× *g* and 4 °C. Subsequently, the aqueous phase was removed, 0.3 mL ethanol was added and the samples were inverted several times, incubated for another 3 min and centrifuged for 5 min at 2000× *g* and 4 °C. The resulting phenol-ethanol supernatant was transferred in new tubes and 1 mL isopropanol was added. The mixture was incubated for 10 min, centrifuged for 10 min at 12,000× *g* and 4 °C to pellet the proteins. The supernatant was discarded. Afterwards, the pellets were washed in 0.2 mL of 0.3 M guanidine hydrochloride buffer in 95% (*v*/*v*) ethanol, incubated for 20 min, and centrifuged for 5 min at 7500× *g* and 4 °C. The supernatant was discarded. Washing steps were repeated 3 times. 2 mL ethanol was added to the protein pellets and mixed by vortexing, followed by incubation for 20 min, and centrifugation for 5 min at 7500× *g* and 4 °C. The supernatant was discarded and the pellets were air-dried. To solve the pellet 200 µL of 1% (*v*/*v*) SDS buffer was added. To ensure complete resuspension, the pellet was incubated for 3 min at 50 °C, and finally centrifuged for 10 min at 10,000× *g* and 4 °C. The resulting protein extract was transferred to a new tube. Protein concentration was measured by Pierce BCA Protein Assay [40,41,42,43].

A quantity of 30 µg of the protein extract was separated on a 4–20% Criterion TGX precast Gel (BioRad, Hercules, CA, USA) and stained with Colloidal Coomassie Brilliant Blue G-250 as published by Neuhoff et al. [44]. Afterwards, each lane was cut in 10 pieces. Each piece was sliced into smaller blocks for tryptic digestion. To this end, gel blocks were destained using a washing solution containing 200 mM ammonium bicarbonate in 30% (*v*/*v*) acetonitrile, incubated in a thermo shaker for 15 min, at 900 rpm and 37 °C. The washing solution was discarded from the tube and the washing step repeated two times. Following destaining, the gel pieces were shrunk to dryness using a vacuum centrifuge. 100 µL of a 2 µg/µL trypsin stock solution (Promega, Fitchburg, WI, USA) was added to the dried gel-pieces and incubated for 15 min at RT. Excess trypsin solution was removed and samples incubated overnight at 37 °C. Subsequently, the gel blocks were covered with Aqua Bidest and incubated in an ultrasonic bath for 15 min to elute the peptides from the gel blocks. The peptide containing supernatant was transferred to new pre-lubricated reaction tubes and desalted using ZipTip purification (C18, Merck Millipore, Billerica, MA, USA) according to the manufacturer’s protocol. Afterwards, the peptide mixture was eluted in glass vials, vacuum centrifuged to dryness, resuspended 10 µL in 0.1% (*v*/*v*) formic acid, and stored at −20 °C.

### 2.5. Mass Spectrometry Analysis

The peptide containing solution was applied to an Easy-nLC II with self- packed RP C18 separation column (100 µm i.d × 200 mm length) [45] for reversed-phase chromatography (Thermo Fisher Scientific, Waltham, MA, USA). Peptides were eluted by a binary gradient of buffers A (0.1% (*v*/*v*)) acetic acid and B (99,9% (*v*/*v*) ACN, 0,1% (*v*/*v*) acetic acid) over a time of 100 min at 300 nL/min, after loading and desalting them on the column. During analysis injection of the peptide mixture was kept constant. Measurements were performed with an LTQ-Orbitrap-Velos mass spectrometer (Thermo Fisher Scientific, Bremen, Germany) equipped with a nanoelectrospray ion source coupled on-line to the chromatographic system. Samples were measured in data-dependent manner with repeated cycles of overview scans in the Orbitrap (r 30,000) with the lock-mass option enabled, followed by MS/MS acquisition of the 20 most intensive precursor ions in the linear ion trap. Dynamic exclusion was enabled [46]. The mass spectrometry proteomics data have been deposited to the ProteomeXchange Consortium via the PRIDE [47] partner repository with the dataset identifier PXD020775.

### 2.6. Database Assembly and Data Analysis

For protein identification, a specific database for the gastrointestinal microbiome of swine was constructed, based on phylogenetic data obtained from 16S rRNA gene sequencing (described above). To this end, all available protein entries of the identified families were downloaded from Uniprot (version 19.11.18). To remove full-length sequence redundancy, the fasta toolkit (fastatk, version 3.0.1, https://gitlab.com/s.fuchs/fastatk) was used. The final database contained 29,366,730 non-redundant sequence entries. For protein identification raw files were searched against the specific database using the Mascot Daemon version 2.6.0 (Matrix Science Ltd., London, UK) with the following search parameters: enzyme: trypsin; variable modifications, methionine oxidation (+15.99 Da); maximal missed cleavages: 2; peptide charge: 2+, 3+ and 4+; peptide tolerance 10 ppm; MS/MS tolerance 0.5 Da. To validate the data, all dat files were applied to Scaffold software version 4.8.7 (Proteome Software Inc., Portland, OR, USA) with the LFDR scoring system and standard protein grouping. Furthermore, X!Tandem was used with a variable modification of methionine (+15.99 Da). Afterwards, a protein threshold of 95% and 1 peptide per protein were applied to the data. Proteins were considered as identified if they were present in at least two out of the two or three biological replicates. If identical peptides occur in different proteins and these proteins could not be discriminated by the MS/MS analysis they were assigned to one protein group.

### 2.7. Taxonomic, Functional and Statistical Analysis of Protein Groups

Taxonomic and functional protein analysis was performed using the metaproteome annotation pipeline Prophane (www.prophane.de). For taxonomic analysis, Prophane used diamond blast combined with the NCBI nr protein database (version 08.08.2018) with an e-value of 0.01, query-coverage of 0.9, and max-target-seqs of 1. For the comparison of protein and 16S rRNA derivated taxonomic classification data, NCBI taxonomy was manually transcribed to SILVA taxonomy. Functional annotation was performed using the hmmscan algorithm combined with TIGRFAMs (version 16.09.2014) and eggNOG database (version 4.5.1) applying an e-value of 0.01. For statistical analysis, data was z transformed and a one-sample ANOVA test was performed (*p*-value 0.05) using MeV [48].

### 2.8. Extraction of Metabolites

100 mg of frozen feces powder was transferred in a FastPrep™ tube (containing lysing matrix E (MP Biomedicals™, Eschwege, Germany) and 2 mL 80% methanol and 400 µL internal standard was added. Powder was disrupted two times for 40 s with 5.5 m/s followed by centrifugation (12,400× *g*, 10 min, 4 °C). The supernatant was transferred into a new tube on ice and the FastPrep™ treatment was repeated once with ice-cold water and 500 µL dichloromethane and once with 2 mL ice-cold water. All supernatants were combined, vortexed, and stored for 10 min on ice. After centrifugation, the water/methanol containing phase was lyophilized. The lyophilized samples were resuspended in 1 mL water and split into two samples for subsequent GC-MS and ^1^H-NMR analysis.

### 2.9. Analysis of Metabolites Using GC-MS and ^1^H-NMR

For GC-MS analysis, the lyophilized extracts were derivatized and measured as previously described in Schultz et al., 2017 [49]. Details on GC-MS parameters are described in Dörries et al., 2014 [50]. Metabolites were absolutely quantified using calibration curves from standards or relative amounts were calculated using ribitol as internal standard with ChromaTOF^®^ V4.508.0 software. Metabolite identification was done by using standard compounds. For ^1^H-NMR analysis, the samples were resuspended in 500 µL PBS, vortexed, and metabolites in the supernatant were measured with a Bruker^®^ Avance II 600 NMR spectrometer according to Schultz et al., 2017 [49]. In total, 70 different metabolites per fecal sample and time point (Figure S1) were detected by standard compounds.

### 2.10. Swine Feces Processing Pipeline Suitable for Multi-Omics Analyses

We compiled several individual steps from the above described methods into a feces processing pipeline suitable for multi-omics analyses (Figure 1). We highlight several of the important steps and considerations. The fecal samples were snap-frozen on dry ice immediately after collection and kept frozen at −80 °C until homogenization and extraction to prevent metabolite and protein degradation. Especially for metabolites, but also for metaproteome analysis, an immediate quenching of protein biosynthesis and metabolism as well as constant storage at −80 °C is of utmost importance [51,52,53]. Moreover, swine feces appear to be a highly heterogeneous sample matrix, e.g., due to incorporated indigestible fibers, which can affect the local composition of the microbiome. Therefore, proper feces homogenization prior to extraction of DNA, proteins and metabolites is crucial for generating representative data sets of microbiome composition and functionality. Different methods for homogenization were tested including (i) grinding of frozen samples with mortar and pestle, (ii) suspending samples in water, (iii) grinding thawed samples via bead mill, as well as (iv) pulverization of frozen samples via CryoPrep (data not shown). Due to the practicability of the tested homogenization methods, the best results were achieved employing the CryoPrep protocol as described in previous studies with different sample materials [54]. 

## 3. Results and Discussion

### 3.1. Multi-Omics Analyses of Swine Feces

The established protocol (see above Section 2.10) enables an effective, contamination-free homogenization of frozen fecal samples for subsequent integrated analysis of 16S rRNA gene sequencing, metaproteomics and metabolomics (Figure 1). Notably, only 300 mg of homogenized feces were required for this approach.

16S rRNA gene analysis. DNA from homogenized feces was extracted using an extraction protocol based on bead beating and phenol/chloroform [33], yielding approx. 130 µg DNA/g feces. TRIzol-based extraction resulted in much lower DNA yields (Table S1). Illumina Miseq amplicon sequencing of the V4 region of the 16S rRNA gene resulted in overall 1040 unique bacterial and archaeal ASVs, with rather uniform richness dynamics over time and a remarkably low variability between the individual fecal microbiomes (Table 2).

Notably, although richness and diversity were found to be highest on day 25, these parameters stayed relatively constant during the study period. The protocol resulted in an observed average richness of the samples of 820 ASVs (±105 ASVs, CV = 0.13) an average Shannon index of 5.17 (±0.15, CV = 0.028) and an average Evenness of 0.77 (±0.011, CV = 0.015). These values were similar or higher as compared to other findings from swine fecal microbiomes [19]. Applying rectal swabs in a longitudinal experiment over 174 days, Wang and colleagues [55] detected up to 500 phylotypes. A fluctuation in richness was observed that depended on dietary changes. A meta-study of microbiomes from porcine GI tract and fecal samples identified 558 core OTUs [20]. This indicates a comparable microbiome diversity obtained with the presented multi-omics sample processing approach.

Metaproteomics: Initially, different extraction protocols, including a chloroform-based protocol for sewage sludge [56], an urea/thiourea-based protocol for microbial biofilms [46], a protocol combining heating and bead-beating [57], and a TRIzol-based protocol, were tested for efficiency (Table S2). The TRIzol-based protocol appeared to be most promising yielding approximately 8 mg protein per g feces on average and resulting in a mean of 4189 (±401) protein groups (PGs) identified in 2 of 2 or 3 biological replicates for each individual sample consisting of 100 mg feces (Table 3). The large majority of the identified PGs were of bacterial origin (average 91%), followed by eukaryotic PGs (Table S3). The number of identified PGs is significantly higher than in other comparable studies on fecal samples (e.g., [11,25,27]) although the processed amount of biomass was lower. This might be explained by the use of 10 fractions instead of 1 or 3 fractions from one gel band after 1D SDS PAGE for mass spectrometry analysis. Tröscher-Mußotter and colleagues [27] identified a mean of 1780 PGs (±153) from 5 g of colon digesta. The study of Heinritz and colleagues [11] resulted in 500 to 740 PG identifications from 300 mg of swine feces. In an integrated multi-omics study of Heintz-Buschart and colleagues [25], focusing on the intestinal microbiome of humans with type 1 diabetes, a mean of 2573 (±1645) PGs has been identified from 200 mg stool samples.

Metabolomics: The homogenous fecal powder was applied to a FastPrep cell disruption followed by water/methanol/dichloromethane metabolite extraction. Different amounts of extraction volume and lysing matrices were tested. The best results were obtained using lysing matrix E and a total extraction volume of 6.5 mL water/methanol/dichloromethane (data not shown). In total, 70 different metabolites per fecal sample and time point (Figure S1) were detected, which is in good accordance with other studies focusing on feces analysis by GC-MS and NMR analysis [51,58,59]. We focused on primary metabolites, i.e., amino acids and related degradation intermediates, carbon core metabolites, fatty acids, and SCFA (Table 4). These metabolites, mainly SCFA, were also analyzed in other studies focusing on swine fecal metabolome [60,61,62].

Compared to other meta-omics studies, in which the amount of samples ranged from 150 mg to 5 g [11,25,27,63,64], our protocol is based on a rather small amount of sample material, i.e., a minimum of 40, 80, and 100 mg for 16S rRNA gene sequencing, metaproteomics, and metabolomics, respectively, enabling integrated multi-omics analyses of low biomass samples.

### 3.2. Taxonomic Composition of the Intestinal Microbiome

**Figure 2:**
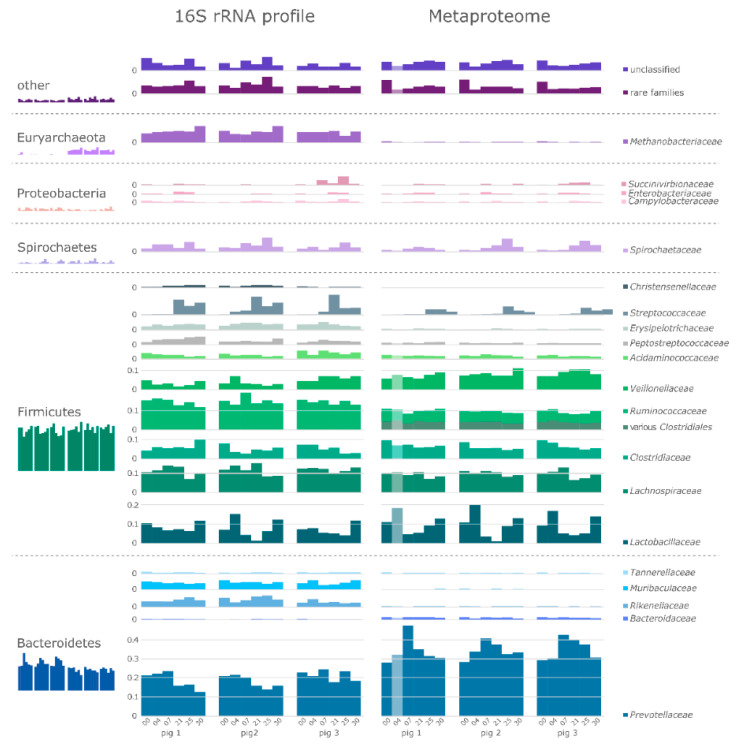
Dynamic development of the fecal microbiota from three healthy swine over 30 days. Comparison of the relative abundances based on 16S rRNA gene and metaproteome data on phylum level (small barplots, left) and family level. For better illustration, only families above 1% of the relative amount were shown. SILVA taxonomy was used for this comparison.

**Figure 3:**
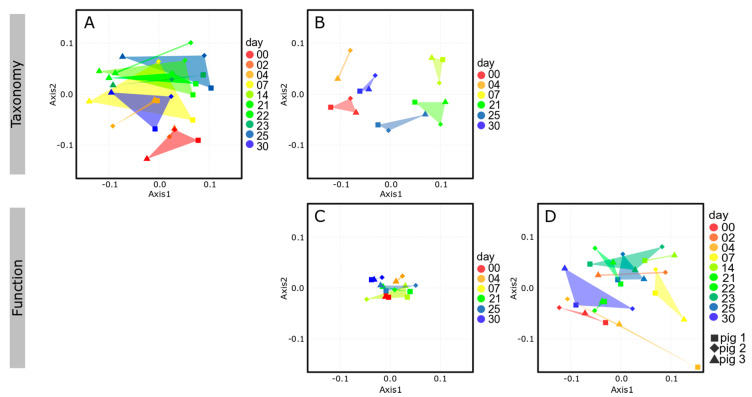
Nonmetric multidimensional scaling (NMDS) plots, based on Bray–Curtis dissimilarities, of the 16S rRNA gene profile (**A**), metaproteome taxonomy (**B**), and function (**C**) and the metabolome (**D**). NMDS plots revealed similar tendencies on taxonomic level (**A**,**B**) as well as on functional level (**C**,**D**).

Determination of microbiome composition was performed at 16S rRNA gene level, representing prokaryotes, both active and dormant, and at metaproteome level, indicative of active prokaryotes. In general, 16S rRNA gene and metaproteome profiles were similar and revealed a typical mammalian fecal microbiome consisting primarily of Firmicutes and Bacteroidetes (Figure 2) [19,65]. Furthermore, all three biological replicates at each timepoint were comparable with similar temporal dynamics. We identified the family *Prevotellaceae* as predominant (approx. 20% of 16S rRNA genes and up to ≈30–35% in metaproteome), which was also described by Heinritz and colleagues [11]. Besides *Prevotellaceae* the families *Lactobacillaceae*, *Lachnospiraceae*, *Veillonellaceae,* and *Clostridiaceae* showed similar variation over time based on 16S rRNA gene sequencing and metaproteomic data. Confirming a previous finding [66], we observed a time-dependent decrease of *Lactobacillaceae*. On metaproteome level, the increasing abundances in *Prevotellaceae* at d7, as well as in *Spirochaetaceae* and *Streptococcaceae* on d21 were directly correlated with the decrease in *Lactobacillaceae*. When the *Lactobacillaceae* recovered, the previously mentioned families started to decrease. Generally, the observed longitudinal dynamics in the taxonomic composition of microbiomes of 16S rRNA genes and protein groups were reflected in the nonmetric multidimensional scaling (NMDS) analyses (Figure 3A,B, Table S4). Here, the positions of the intermediate time points (d7-d25) formed clusters in close proximity to each other, and separated from the early (d0 and d2) timepoints (Figure 3A) and from the early (d0 and d4) and late (d30) timepoints (Figure 3B). Temporal dynamics of the active microbiome over a 30-day experiment were expected in the eight weeks old piglets [55], however this intermediate shift in microbiome composition was not anticipated. Interestingly, we detected *Methanobacteriaceae* primarily at 16S rRNA gene level, but with much lower abundance in the metaproteome. This discrepancy might have been caused by primer bias and/or low identification rate for archaeal proteins in the metaproteome analysis. Furthermore, the observed variation can be explained on a biological level, regarding the different copy numbers of 16S rRNA genes in different species [67]. The large difference in the relative abundance of *Ruminococcaceae* between 16S rRNA and the taxonomic metaproteome analysis can be explained by the LCA algorithm used for the taxonomic assignment of the PGs, since many PGs of Clostridiales cannot be reliably classified on family level as *Ruminococcaceae*, thus these PGs remain classified as ‘various’. Nevertheless, the described taxonomic similarities between 16S rRNA gene and metaproteome level underline the reliability of the presented approach. Nevertheless, the described taxonomic similarities between 16S rRNA gene and metaproteome level, e.g., the appearance of *Streptococcaceae* at day 21, and the time dependent variation of *Lactobacillaceae* and *Spirochaetaceae* abundance underline the reliability of the presented approach.

#### High Similarity of the Fecal and Colonal Microbiota

Using 16S rRNA gene amplicon sequencing, we tested if the microbiome composition of feces can serve as a reliable minimal-invasive proxy for the intestinal microbiome composition. Looft and colleagues indicated limited transferability of fecal microbiomes compared to microbiomes of the intestinal tract, due to the low number of reference studies [68].

**Figure 4:**
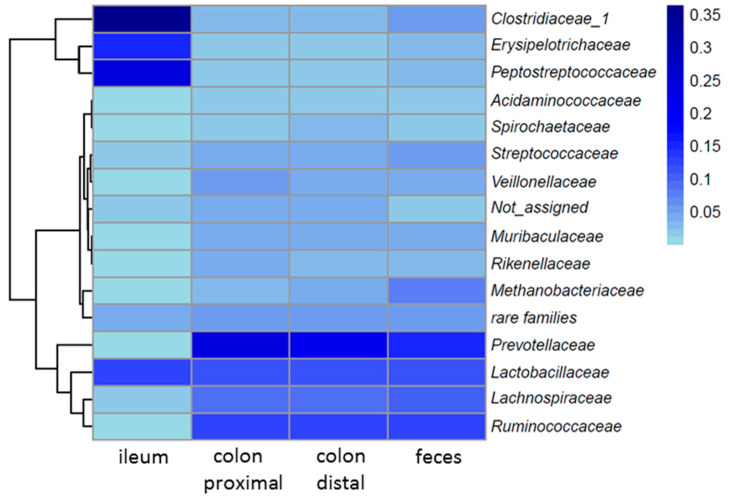
Fecal community of swine 1, 2, and 3 (merged) composition and comparison between the intestinal samples on family level. Color code from light blue to dark blue indicates an increasing relative abundance.

We compared microbiomes from feces with the corresponding microbiomes from distal and proximal colon lumen, and from ileum (Figure 4, Table S5). While we saw a high accordance of the two colonal microbiomes with the fecal microbiome (without significant differences), the microbiome from ileal samples differed strongly (e.g., ileum vs. proximal colon *p* = 0.006). Furthermore, richness data indicate a high significant difference between the merged fecal- and colonal richness and the one from ileum (*p* = 0.001). The large intestine is primarily dominated by *Prevotellaceae*, *Ruminococcaceae*, *Lachnospiraceae*, and *Lactobacillaceae*, whereas the most abundant families in the ileum are *Clostridiaceae*, *Erysipelotrichaceae* and *Peptostreptococcaceae*. This is likely due to the higher transition rates in the ileum. In the study of Deusch and colleagues [69] the fecal microbiome was also compared to intestinal microbiomes (ileum and colon) of livestock. Similar to our results, the colonal and fecal microbiomes showed a more similar composition than with the ileal microbiomes. The opportunity of taking feces as proxy for the large intestine could pave the way for (simple) predictions of the intestinal status with “minimal-invasive“ sampling techniques, thus enabling follow-up of the same individuals over a longer period of time without the need for additional animals that have to be sacrificed during the experimental trial.

### 3.3. Functional Analysis of the Microbiome

**Figure 5:**
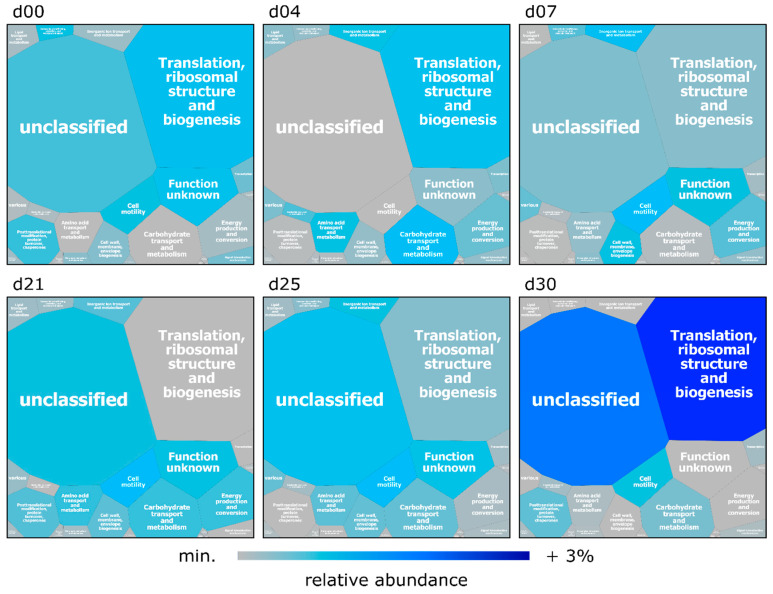
Voronoi Treemap illustrating functions (based on eggNOG categories) of the identified PGs from swine feces collected over 30 days. Field size represents the average over all samples minimal relative abundance over time of the corresponding category. The grey color represents the minimum expression of a functional category; dark blue fields indicate at maximum an increase of 3% in the relative abundance of the PGs at the sampling days compared to the minimum.

Functional assignment of the identified PGs (see Materials and Methods) revealed a similar distribution for all metaproteomes at the qualitative level during the entire course of the experiment (Figure 5). More than 65% of the identified PGs were assigned to specific biological functions, which is in good accordance to the ratio of described functional annotations of the employed eggNOG database [70]. A proportion ranging from 25% to 30% of the PGs were involved in protein biosynthesis (“translation, ribosomal structure and biogenesis”) with the most prominent ribosomal proteins L and S originating from the bacterial families *Prevotellaceae*, *Clostridiaceae*, *Lactobacillaceae*, *Lachnospiraceae*, *Ruminococcaceae,* and *Selenomonadaceae*. Other abundant PGs belonged to the category “carbohydrate transport and metabolism” (3–7%), among them many proteins involved in glycolysis and gluconeogenesis, and “energy production and conversion” (3–5%), including proteins linked to the ATP synthase and NADH dehydrogenase complex. Moreover, proteins belonging to the functional category “post-translational modification, protein turnover, chaperones” (3–4%), “cell motility” (2–4%) and “amino acid transport and metabolism” (2–3%) were frequently identified (Figure 5, Figure S2). A detailed list of all and frequently found PGs, their taxonomical origin and functional assignment are provided in Tables S6–S8, respectively. In contrast to the time-related dynamics in the taxonomic composition of the microbiome, we observed overlaps of the NMDS clusters based on functional protein annotations (Figure 3C). In conclusion, our data suggest that albeit the taxonomic composition of the microbiome alters along the time course of the experiment, its functional potential remains rather stable compared to the taxonomy (Figure 5, Figure S2), indicating a functional redundancy among microbiome members, as proposed by others (e.g., [11]).

To identify important metabolites and to complement the metaproteomics dataset, metabolome analyses were performed. Microbial metabolites are known to affect host metabolism [71,72] and immune system [73,74,75,76] and can thus play a significant role in the health status of the host. Using ^1^H-NMR and GC-MS 70 different metabolites were identified (Table 4). These cover a broad spectrum of metabolic intermediates, production of which seemed to be triggered by the complex host-microbiota interplay. Among the frequently identified compounds were: metabolites of the central carbon metabolism (e.g., glucose, succinate or malate), amino acids (e.g., lysine and valine, aspartate, glutamate, glycine and tyrosine), as well as palmitate and 3-hydroxybutyrate. Lysine and valine were described as preferred substrates for gut bacteria [77]. In addition, large quantities of ornithine, methionine and arginine were found, which could be catabolized in polyamines [78]. Polyamines are involved in enhancing the integrity of the intestinal barrier and are responsible for maintaining, rapid turnover and proliferation of intestinal epithelial cells. Apart from this, polyamines stimulate the production of intercellular junction proteins, which regulate the epithelial barrier function cells [79]. Interestingly, Löser and colleagues [80] have demonstrated an important role of intestinal polyamine pools during the postnatal development of the gastrointestinal tract.

Metabolite concentrations determined from feces can be regarded as rough estimation but they do not reflect the exact metabolite levels present in the colon [81]. Due to the dynamic nature of metabolites and the fact that their production is affected by internal and external factors (e.g., enzyme activity, storage or analysis method) [82], metabolome analysis revealed larger differences between the individual animals and sampling days compared to the consistent profile of functional metaproteome (Figure 3C,D). Visual overlay of metaproteomics and metabolomics data provided a detailed overview of the metabolic activities in the microbiome (Figure S3). Remarkably, nearly all metabolites could be matched to pathways that were postulated to be active based on our metaproteomic data using the online tool IPath3 [83].

**Figure 6:**
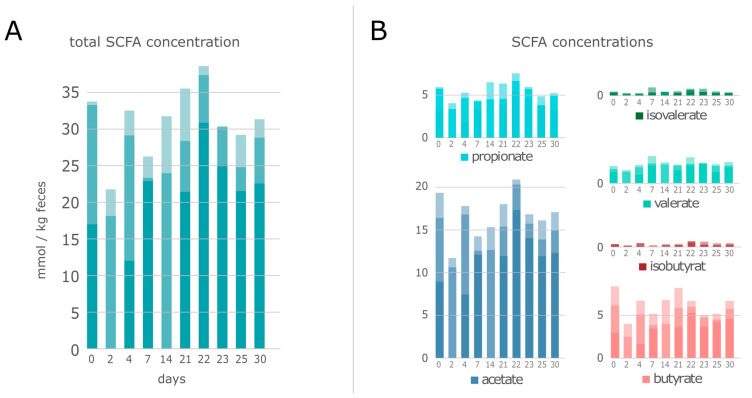
Total amount of all SCFAs (**A**) and concentrations of acetate, propionate, butyrate, valerate, isobutyrate, and isovalerate (**B**) of swine fecal samples from ^1^H-NMR analysis over 30 days. Y-axes: SCFA concentration in mmol/kg feces; X-axes: sampling day.

SCFAs, central metabolites that are exclusively synthesized by different microbiome members, are known to affect the health status of the host (reviewed in, e.g., Koh et al. [72]). They have been implicated in host cell proliferation, epithelial cell integrity, histone (de-)acetylation, and G-protein coupled receptor 43 (GPR43) activation (reviewed in, e.g., Venegas et al. [84]). Our metabolome analysis revealed acetate as the most abundant SCFA, which has been also observed in various other studies (e.g., [11,23,78]). Furthermore, butyrate and propionate were detected in high concentrations (Figure 6). The detected amounts of SCFAs were comparable to a study by Heinritz and colleagues, that investigated the effects of different diets on the microbiome composition and metabolite production in 12-week-old pigs [11]. There were several studies [85,86,87,88] that measured higher SCFA concentrations in gastrointestinal tract samples from pigs. However, it is important to note that some of these studies [85,87,88] used digesta samples from different gastrointestinal sections instead of fecal samples for SCFA measurement. It was recently shown that fecal samples have lower concentrations of SCFAs, due to the fact that 95 % of the produced SCFAs were absorbed during the flow of contents through the gastrointestinal tract. Therefore, feces were known to have lower concentrations of SCFAs than the digesta of intestinal sections [89].

In good accordance to the metabolome data, our metaproteomics study identified several key-enzymes of the SCFA production pathways, i.e., acetyl-CoA acetyltransferase, acetyl-CoA carboxylase, methylmalonyl-CoA mutase, butyrate kinase, and acetate kinase. These enzymes were expressed by different members of the orders Clostridiales, Bacteroidales, Selenomonadales, Veillonellales, and Acidaminococcales. Especially the orders Clostridiales (e.g., *Lachnospiraceae* and *Ruminococcaceae*) and Bacteroidales (e.g., *Bacteroidaceae* and *Prevotellaceae*) were known as main SCFA producers in the gut [20,72]. Albeit the amounts of acetate, propionate, butyrate and valerate reached a slight maximum at day 22 before decreasing, rather stable SCFA concentrations were observed during 30 days (Figure 6). Notably, we detected an increased abundance of the families *Streptococcaceae* and *Spirochaetaceae* while acetate and propionate were measured in higher concentrations. 

## 4. Conclusions

In conclusion, we developed an integrated multi-omics protocol for the analysis of porcine feces, which enabled a contact-free, high-throughput homogenization of the heterogenous fecal sample, while keeping it frozen. The homogeneous fecal powder was easily divided for the use of specialized extraction protocols for the individual omics techniques. Furthermore, we found that the taxonomic composition of feces and colon rather congruent. Therefore, we suggest that feces could be used as minimal-invasive proxy for the colon in future analyses of the gastrointestinal tract.

Applying this protocol in a typical biomedical experiment revealed congruent dynamics in microbiome structure and function but also large conformity between the different pigs. Taxonomic analysis showed a dynamic microbiome composition, which is not surprising given the successional dynamics of eight-week-old piglets over 30 days. Consequently, these high microbiome dynamics have to be considered in the design of biomedical experiments using swine.

Functional analysis via metaproteomics revealed low variability between the individual animals and at the sampling days. Through this observation, infection-related changes in the microbiome could be detected in further experiments. Integration of metaproteomic and metabolomic data resulted in large coverage of the metabolic pathways. Because main functional assignments and metabolites, such as SCFAs, were more or less stable, we suggest, that these findings point towards functional redundancy during the natural development of the individual intestinal microbiota. To verify this hypothesis, we will analyze the influence of time and infection on the functional potential of the gastrointestinal microbiome of swine in further infection studies.

Our study and the resulting multi-omics sample processing pipeline can pave the way for a more detailed analysis in the context of future porcine infection experiments with swine pathogenic microorganisms, e.g., influenza A virus or *Streptococcus suis*, as well as any other event that alters the gastrointestinal microbiome (e.g., after weaning or dietary changes). Further improvements could be beneficial for the presented approach, such as the use of additional replicates, to enable more robust statistical analyses. Furthermore, the use of metagenomics or transcriptomics instead of 16S rRNA genes to create ecosystem-specific databases for the analysis of the metaproteome would likely increase the number of identified protein groups, and might also reduce inconsistencies in the taxonomic classification between nucleic acid and protein group based profiles.

## Figures and Tables

**Table 1:** Longitudinal sampling scheme and number of samples at each sampling day (d) for meta-omics analysis. Individual samples were collected from three healthy animals.

		Days after Starting Point									
		0	2 *	4 **	7	14 *	21	22	23	25	30
Fecal samples (*n* = 3)		3	2	3	3	2	3	3	3	3	3
Individual number of analyzed samples after homogenization	16S rRNA gene analysis	3	2	3	3	2	3	3	3	3	3
	Metaproteomics	3	-	2	3	-	3	-	-	3	3
	Metabolomics	3	2	3	3	2	3	3	3	3	3

* only two samples were available. ** low amount of sample material from one animal.

**Table 2:** Microbiome alpha-diversity parameters. Average, standard deviation and coefficient of variance (CV) of richness (number of ASVs), Shannon, and Simpson index of the fecal microbiota sampled from healthy piglets along the experiment.

	Days after Sampling									
	0	2	4 *	7	14 *	21	22	23	25	30
No. of ASVs ± sd; CV	831 ± 97; 0.12	716 ± 112; 0.16	773 ± 36; 0.05	816 ± 169; 0.21	859 ± 161; 0.19	841 ± 88; 0.1	828 ± 80; 0.1	857 ± 161; 0.19	901 ± 99; 0.11	780 ± 47; 0.06
Shannon ± sd; CV	5.19 ± 0.10; 0.02	5.00 ± 0.12; 0.02	5.10 ± 0.13; 0.02	5.30 ± 0.26; 0.05	5.26 ± 0.23; 0.04	5.14 ± 0.17; 0.03	5.13 ± 0.13; 0.03	5.23 ± 0.13; 0.03	5.40 ± 0.14; 0.03	5.02 ± 0.05; 0.01
Simpson ± sd; CV	0.99 ± 0.001; 0.001	0.98 ± 0.003; 0.004	0.99 ± 0.002; 0.002	0.99 ± 0.003; 0.003	0.99 ± 0.003; 0.003	0.98 ± 0.003; 0.03	0.98 ± 0.003; 0.003	0.98 ± 0.003; 0.003	0.99 ± 0.001; 0.001	0.98 ± 0.002; 0.002
Evenness ± sd; CV	0.77 ± 0.006; 0.007	0.76 ± 0.001; 0.001	0.77 ± 0.021; 0.027	0.79 ± 0.015; 0.019	0.78 ± 0.013; 0.016	0.76 ± 0.013; 0.017	0.76 ± 0.009; 0.012	0.78 ± 0.016; 0.021	0.79 ± 0.011; 0.013	0.75 ± 0.012; 0.016

* samples from only two individuals were available.

**Table 3:** Protein groups identified from porcine feces including total and unique (per biological replicate) numbers of PGs from individual swine feces samples at the corresponding sampling days as well as numbers of PG identified in at least 2 of 3 biological replicates.

	Days after Sampling					
	0	4 *	7	21	25	30
Pig 1	5228		6215	5038	4224	4886
unique	1091		2653	1279	960	1108
Pig 2	5342	6920	3672	5274	5450	6101
unique	1231	3303	1059	1458	2068	2217
Pig 3	6271	5686	4837	5022	5204	5298
unique	2052	2069	1539	1601	1842	1289
In 2 of 3	4797	3617	3882	4380	3970	4491

* only two biological replicates available.

**Table 4:** Classification of detected metabolites into main metabolic pathways.

Pathway	Number of Detected Metabolites
carbon core metabolism	13
amino acids	18
amino acid degradation	6
fatty acids	6
short-chain fatty acids	6
various	21
∑	70

## Supplementary Materials

The following are available online at https://www.mdpi.com/2076-2607/8/12/1887/s1. Figure S1: Heatmap displaying fold-change (mean amount day x/mean amount over 30 days) of detected metabolites in pig feces by GC-MS and ^1^H-NMR spectroscopy. Dark blue color indicates an increased fold change of 2.5, while white color represents a decrease in the fold change to 0.3. Figure S2: Assignment of identified PGs from swine feces to functional categories based on eggnog (n = 3 for sampling day 0, 7, 21, 25 and 30, n = 2 for sampling day 4). For the functional analysis of the metaproteome all identified PGs were considered, including unclassified PGs and PGs with unknown function. Figure S3: Integration of metaproteome and metabolite profiles of swine feces. Blue lines highlight the identified proteins of corresponding pathways. Detected metabolites were grouped in the categories SCFAs (teal), fatty acids (dark green), carbon core metabolism (purple), amino acids (orange), amino acid degradation (yellow), various (black), and depicted as spots. Table S1: Amount of isolated DNA from swine feces using different extraction protocols. Table S2: Average protein concentration from swine feces after employing different protein extraction protocols. Table S3: Total number of identified protein groups from swine feces at the corresponding sampling days. Table S4: nmds data 16S metaprot metabolome. Table S5: data heatmap intestinals. Table S6: frequent families. Table S7: frequent proteins. Table S8: prophane output all protein groups.
