# Supplementary material for: A Multi-Omics Protocol for Swine Feces to Elucidate Longitudinal Dynamics in Microbiome Structure and Function
Source: Microorganisms. 2020 Nov 28;8(12):1887. doi: 10.3390/microorganisms8121887 (PMC7760263; doi:10.3390/microorganisms8121887)
Supplement: Supplementary file 1 [file microorganisms-08-01887-s001.zip › microorganisms-939054-supplementary.pdf]

Supplementary materials

An integrated multi-omics protocol for swine feces reveals congruent longitudinal dynamics in microbiome structure and function

A Multi-Omics Protocol for Swine Feces to Elucidate Longitudinal Dynamics in Microbiome Structure and Function

Laurin Christopher Gierse [1,†], Alexander Meene [1,†], Daniel Schultz [2], Theresa Schwaiger [3], Claudia Karte [3],
Charlotte Schröder [3], Haitao Wang [1], Christine Wünsche [1], Karen Methling [2], Bernd Kreikemeyer [4], Stephan Fuchs [5], Jörg Bernhardt [1], Dörte Becher [1], Michael Lalk [2], KoInfekt Study Group [‡], Tim Urich [1,*] and Katharina Riedel [1,*]

[1] Institute of Microbiology, University of Greifswald, Felix-Hausdorff-Str. 8, 17489 Greifswald, Germany; laurin.gierse@uni-greifswald.de (L.G.); alexander.meene@uni-greifswald.de (A.M.); haitao.wang@uni-greifswald.de (H.W.), christine.wuensche@uni-greifswald.de (C.W.); joerg.bernhardt@uni-greifswald.de (J.B.); doerte.becher@uni-greifswald.de (D.B.)

[2] Institute of Biochemistry, University of Greifswald, Felix-Hausdorff-Str. 4, 17489 Greifswald, Germany; daniel.schultz@uni-greifswald.de (D.S.); methling@uni-greifswald.de (K.M.); lalk@uni-greifswald.de (M.L.)

[3] Friedrich-Loeffler-Institut, Greifswald-Insel Riems, Südufer 10, 17493 Greifswald, Germany; theresa.schwaiger@boehringer-ingelheim.com (T.S.); claudia.karte@fli.de (C.K.); charlotte.schroeder@fli.de (C.S.)

[4] Institute for Medical Microbiology, Virology and Hygiene, Rostock University Medical Centre, Schillingallee 70, 18055 Rostock, Germany; bernd.kreikemeyer@med.uni-rostock.de

[5] Division of Nosocomial Pathogens and Antibiotic Resistance, Robert Koch Institute Wernigerode, Burgstraße 37, 38855 Wernigerode, Germany; fuchss@rki.de

* Correspondence: tim.urich@uni-greifswald.de (T.U.); riedela@uni-greifswald.de (K.R.); Tel.: +49-383-442-059-04 (T.U.); +49-383-442-059-00 (K.R.)

† These authors contributed equally to the work.

‡ Authors of the KoInfekt Study Group were listed in the Author Contributions

Received: 4 September 2020; Accepted: 27 November 2020; Published: 28 November 2020

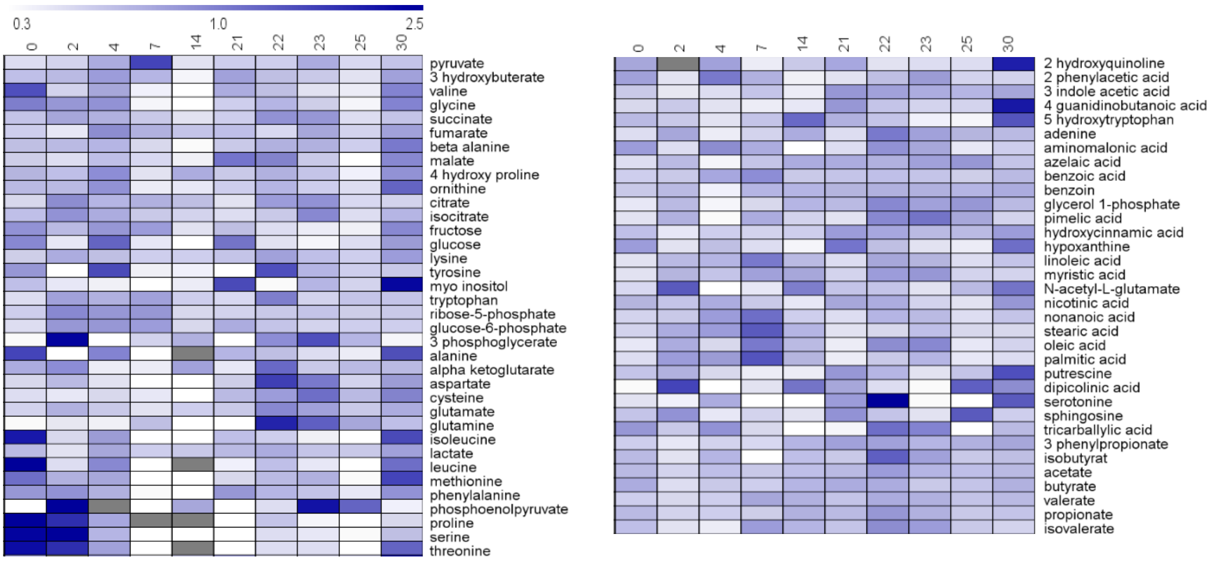

Figure S1: Heatmap displaying fold-change (mean amount day x / mean amount over 30 days) of detected metabolites in pig feces by GC-MS and ¹H-NMR spectroscopy. Dark blue color indicates an increased fold change of 2.5, while white color represents a decrease in the fold change to 0.3.

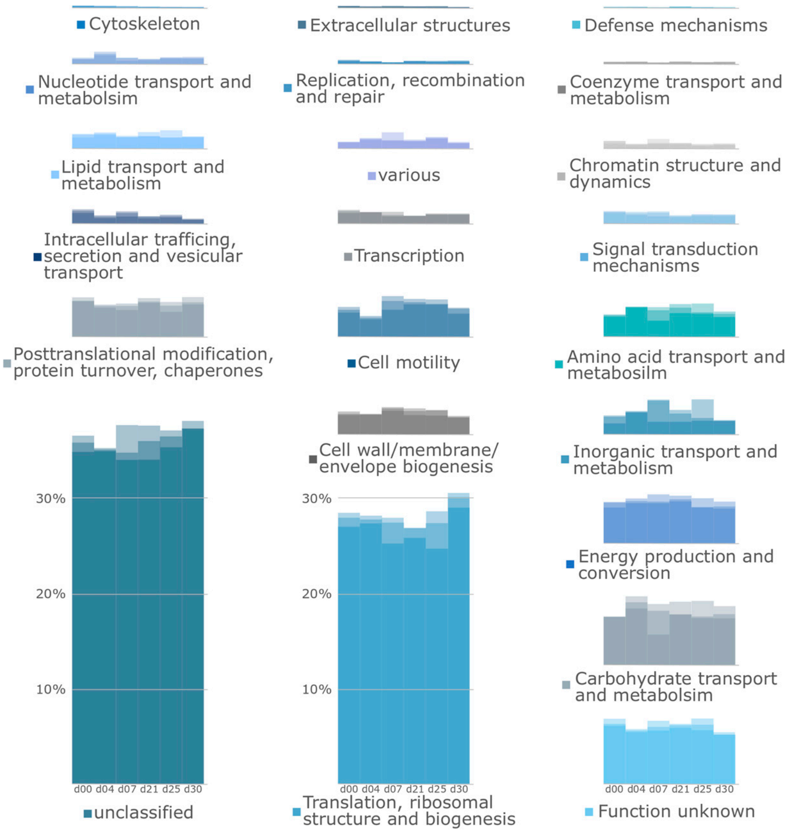

Figure S2: Assignment of identified PGs from swine feces to functional categories based on eggnog (n = 3 for sampling day 0, 7, 21, 25 and 30, n = 2 for sampling day 4). For the functional analysis of the metaproteome all identified PGs were considered, including unclassified PGs and PGs with unknown function.

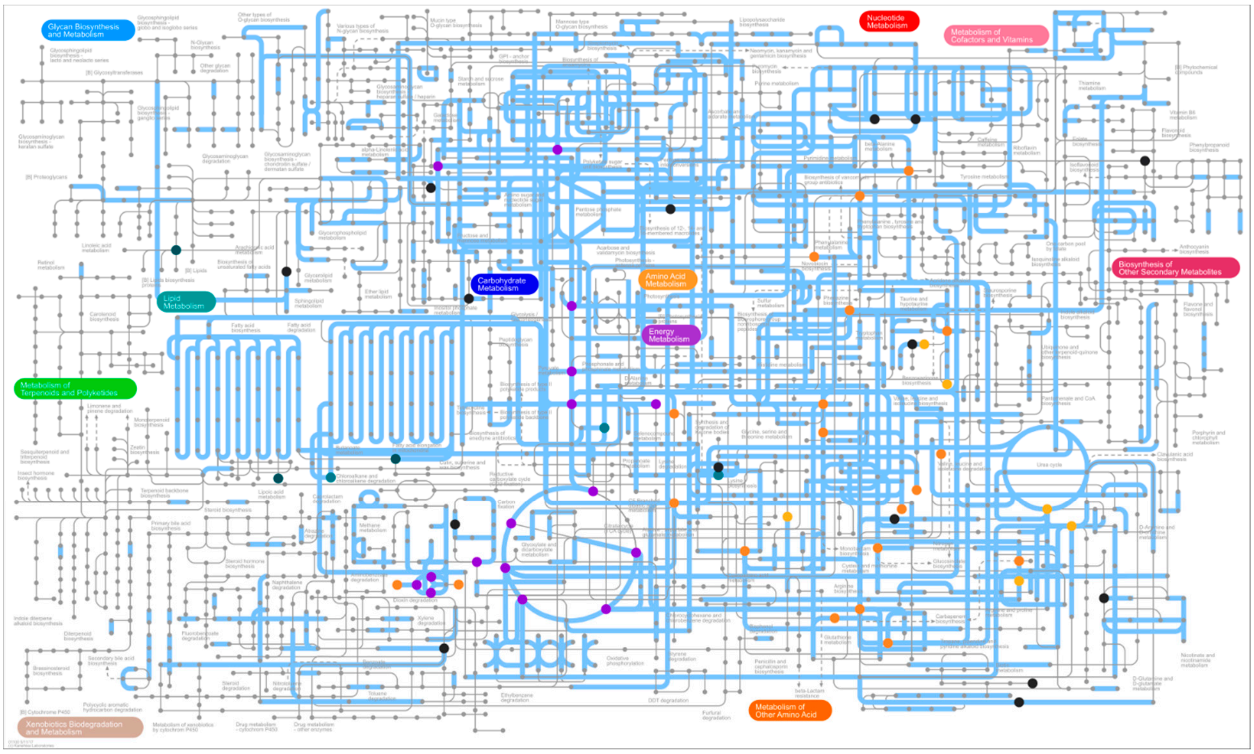

Figure S3: Integration of metaproteome and metabolite profiles of swine feces. Blue lines highlight the identified proteins of corresponding pathways. Detected metabolites were grouped in the categories SCFAs (teal), fatty acids (dark green), carbon core metabolism (purple), amino acids (orange), amino acid degradation (yellow), various (black), and depicted as spots.

Table S1: Amount of isolated DNA from swine feces using different extraction protocols.

	tested extraction protocols for 16S rRNA sequencing	
	TRIzol-based	phenol-chloroform
**amount DNA [µg /g feces]**	10.68	50.11
**SD [µg / g feces]**	0.52	2.16

Table S2: Average protein concentration from swine feces after employing different protein extraction protocols.

	tested extraction protocols for metaproteomics			
	sewage sludge	urea/thiourea	heating and bead-beating	TRIzol-based
**amount protein [µg / µl]**	4.67	1.27	1.64	5.76
**SD [µg / µl]**	0.57	0.21	0.14	0.39

Table S3: Total number of identified protein groups from swine feces at the corresponding sampling days.

	days after starting point					
	**0**	**4***	**7**	**21**	**25**	**30**
**Total**	4560	3454	3705	4230	3817	4348
Bacteria	4062	3194	3386	3924	3506	4055
Eukaryota	403	214	267	228	234	223
Archaea	31	11	4	10	12	20
Various	52	28	37	56	54	34
Unclassified	12	7	11	12	11	16

*only two biological replicates available
